# Breastfeeding and the risk for diarrhea morbidity and mortality

**DOI:** 10.1186/1471-2458-11-S3-S15

**Published:** 2011-04-13

**Authors:** Laura M Lamberti, Christa L Fischer Walker, Adi Noiman, Cesar Victora, Robert E Black

**Affiliations:** 1 Department of International Health, Johns Hopkins Bloomberg School of Public Health, Baltimore, MD, USA; 2 Department of Social Medicine, Federal University of Pelotas, Porto Alegre, RS, Brazil

## Abstract

**Background:**

Lack of exclusive breastfeeding among infants 0-5 months of age and no breastfeeding among children 6-23 months of age are associated with increased diarrhea morbidity and mortality in developing countries. We estimate the protective effects conferred by varying levels of breastfeeding exposure against diarrhea incidence, diarrhea prevalence, diarrhea mortality, all-cause mortality, and hospitalization for diarrhea illness.

**Methods:**

We systematically reviewed all literature published from 1980 to 2009 assessing levels of suboptimal breastfeeding as a risk factor for selected diarrhea morbidity and mortality outcomes. We conducted random effects meta-analyses to generate pooled relative risks by outcome and age category.

**Results:**

We found a large body of evidence for the protective effects of breastfeeding against diarrhea incidence, prevalence, hospitalizations, diarrhea mortality, and all-cause mortality. The results of random effects meta-analyses of eighteen included studies indicated varying degrees of protection across levels of breastfeeding exposure with the greatest protection conferred by exclusive breastfeeding among infants 0-5 months of age and by any breastfeeding among infants and young children 6-23 months of age. Specifically, not breastfeeding resulted in an excess risk of diarrhea mortality in comparison to exclusive breastfeeding among infants 0-5 months of age (RR: 10.52) and to any breastfeeding among children aged 6-23 months (RR: 2.18).

**Conclusions:**

Our findings support the current WHO recommendation for exclusive breastfeeding during the first 6 months of life as a key child survival intervention. Our findings also highlight the importance of breastfeeding to protect against diarrhea-specific morbidity and mortality throughout the first 2 years of life.

## Background

The benefits of breastfeeding on infant and child morbidity and mortality are well documented, with observational studies dating back to the 1960s and 1970s [1-4]. Studies show that human milk glycans, which include oligosaccharides in their free and conjugated forms, are part of a natural immunological mechanism that accounts for the way in which human milk protects breastfed infants against diarrheal disease [5]. In addition, breastfeeding reduces exposure to contaminated fluids and foods, and contributes to ensuring adequate nutrition and thus non-specific immunity. Despite evidence supporting the positive and cost-effective health impacts of exclusive breastfeeding on child survival [6] the practice in resource-poor areas of the world is low. In Africa, Asia, Latin America, and the Caribbean, only 47-57% of infants less than two months and 25-31% of infants 2-5 months are exclusively breastfed, and the proportion of infants 6-11 months of age receiving any breastmilk is even lower [7].

Given that diarrheal disease accounts for approximately 1.34 million deaths among children ages 0-59 months and continues to act as the second leading cause of death in this age group [8], it is important to quantify the preventive effect of breastfeeding practices on diarrhea-specific morbidity and mortality. Very few individual studies have been designed or powered to detect the effects of breastfeeding practices on diarrhea-specific morbidity and mortality for children 0-23 months of age in resource-limited settings.

In 2001, a systematic review of sixteen independent studies conducted by the WHO attempted to resolve the “weanling’s dilemma” in developing countries. The review, which assessed the effects of exclusive breastfeeding for 6 months versus 3-4 months with mixed breastfeeding thereafter, resulted in the recommendation to promote exclusive breastfeeding for the first 6 months of life [9]. More recently, the authors of the Lancet nutrition series published a random effects meta-analysis estimating the increased risk of diarrhea-specific morbidity and mortality among children younger than 2 years in relation to suboptimal breastfeeding practices [7]. While these estimates provide confirmation of the protective effect of breastfeeding, they were based on a limited data set, rather than a complete systematic review, and thus a more thorough and updated revision is warranted.

Building upon previous reviews, this systematic review and meta-analyses use carefully developed and standardized methods to focus on the effects of breastfeeding practices as they relate to diarrhea incidence, prevalence, mortality and hospitalization among children 0-23 months of age. Here we present a comprehensive systematic review and meta-analysis as evidence to be utilized by the Lives Saved Tool (LiST) to model the effect of breastfeeding practices on diarrhea-specific morbidity and mortality [10,11]. The results of our analysis will serve as the basis for generating projections of child lives that could be saved by increasing exclusive breastfeeding until 6 months of age and continued breastfeeding until 23 months of age.

## Methods

We systematically reviewed all literature published from 1980 to 2009 to identify studies with data assessing levels of suboptimal breastfeeding as a risk factor for diarrhea morbidity and mortality outcomes. We conducted our initial search on July 28, 2009 and two updated searches on April 8 and May 5, 2010. All searches were completed in Pubmed, EMBASE, the Global Health Library Global Index and Regional Index, and the Cochrane central register for controlled trials using combinations of key search terms: *breastfeeding*, *breast milk*, *human milk*, *diarrhea*, *gastroenteritis*, *morbidity*, *mortality*, *infant and child*. To ensure the identification of all relevant literature, we also reviewed the references of included papers.

After initially screening for eligibility based on title and abstract, we thoroughly reviewed full publications for inclusion and exclusion criteria outlined *a priori*. We included randomized controlled trials (RCT), cohort and observational studies that assessed suboptimal breastfeeding as a risk factor for at least one of the following outcomes: diarrhea incidence, diarrhea prevalence, diarrhea mortality, all-cause mortality, and diarrhea hospitalizations. Included studies were published in any language from 1980 - 2009 and were conducted in developing countries with a target population of children 0-23 months of age. We excluded studies reporting diarrhea as a result of only one microbial cause, and those with unclear methodology or data in a form that could not be extracted for meta-analysis. We also excluded studies reporting exclusive breastfeeding for children beyond 6 months of age and those failing to restrict the allocation of diarrhea outcomes to *concurrent* breastfeeding status. Additionally, we excluded morbidity studies with diarrhea recall beyond two weeks and mortality studies where the removal of deaths occurring within the first three to seven days of life was not possible. For studies reporting outcomes stratified by HIV status, we only abstracted data on HIV-negative infants and children.

We abstracted data for each diarrhea outcome by breastfeeding exposure levels, which were classified according to current WHO definitions (Table 1) [12,13]. To allow for the comparability of breastfeeding labels and definitions derived from studies published over multiple decades, during which time breastfeeding definitions and terms evolved, we assigned the exposure categories described by each study to a WHO category on the basis of the study’s definition of that exposure category, not the authors’ category label. The majority of discrepancies between breastfeeding label and definition arose over the term ‘exclusive breastfeeding’. By current standards, ‘exclusive breastfeeding’ does not include the ingestion of anything other than breastmilk and prescribed vitamins and medications, and infants receiving non-nutritive liquids, such as waters and teas, are classified as ‘predominantly breastfed’ [12]. This distinction was not formally recommended until 1988 when a meeting of the Interagency Group for Action on Breastfeeding first proposed the development of a set of standardized breastfeeding definitions [14]. WHO officially integrated indicators differentiating between exclusive and predominant breastfeeding in 1991 [12]. As such, for this review we assumed the ‘exclusive breastfeeding’ category was more appropriately labelled ‘predominant breastfeeding’ for studies published prior to 1991, unless the study specifically defined exclusive breastfeeding according to the current definition.

**Table 1:** Breastfeeding exposures

**Exposure Category**[12]	Permitted to Receive
Exclusive Breastfeeding	• breast milk from mother or wet nurse or expressed breast milk • NO other liquids or solids except vitamin drops or syrups, mineral supplements, or prescribed medicines

Predominant Breastfeeding	• breast milk from mother or wet nurse or expressed breast milk • water and water-based drinks • NO food-based fluid with the exception of fruit juice and sugar water • vitamin drops or syrups, mineral supplements, or prescribed medicines

Partial Breastfeeding	• breast milk from mother or wet nurse or expressed breast milk • any other liquids or non-liquids, including both milk and non-milk products

No Breastfeeding	• formula and/or animal’s milk • NO breast milk

Any Breastfeeding	• breast milk from mother or wet nurse or expressed breast milk • Includes children exclusively, predominantly, fully, and partially breastfed

For studies that grouped exclusively and predominantly breastfed infants into a ‘fully breastfeeding’ category, we employed a conservative approach in which fully breastfeeding exposure was treated as predominant. We excluded studies that combined exposures other than exclusive and predominant breastfeeding into one breastfeeding category.

In this review we did not seek to address the issue of early initiation of breastfeeding and prelacteal feeds. Thus, in assigning breastfeeding exposure, we did not differentiate between exclusive and predominant breastfeeding on the basis of receipt of prelacteal feeds during the first 3 days of life.

We extracted effect measures and 95% confidence intervals from all included studies. In cases where relative risk (RR) was not reported, we generated RR and 95% confidence intervals using reported numerators and denominators.

We organized data into the following age strata: 0-28 days, 0-5 mos, 0-11 mos, 6-11 mos, 12-23 mos, and 6-23 mos. We excluded studies with overarching age categories that could not be collapsed; however, we included one diarrhea mortality study grouping children 12-35 mos and applied its RR to the 12-23 mos analysis [15]. For infants aged 0-5 mos, we generated pooled effect measures using exclusive, predominant, and partial breastfeeding as reference categories. For infants in the 0-11 mos category, we used partial and any breastfeeding as reference categories, and for all age categories extending from 6 or 12 months, we used any breastfeeding as the only reference category.

We conducted fixed effects meta-analyses to combine effect measures within a given study that had been reported separately for ages falling within the same category in our analysis. To generate a combined effect measure across studies, we ran a random effects meta-analysis for each comparison. All meta-analyses were performed using the meta command in STATA 10.1 [16].

For each outcome of interest, we summarized the evidence by conducting an assessment of study quality and quantitative measures as per CHERG guidelines. As per the CHERG grading system, the overall quality of evidence for each effect estimate receives a score on a four point continuum (‘high’, ‘moderate’, ‘low’, ‘very low’), which is then used to either support or oppose its inclusion in the LiST model [11]. To further evaluate the limitations of included studies, we created a scoring system to assess the degree to which studies had accounted for reverse causality and self-selection—two major forms of bias in assessing the association between breastfeeding and diarrhea morbidity and mortality. Reverse causation bias results when breastfeeding cessation is a direct consequence of diarrheal illness. Self-selection bias occurs when children are weaned because they became repeatedly ill or grew improperly while breastfed. Although, it has been reported that self-selection or reverse causation can also create bias in the opposite direction, with some mothers less likely to wean sick children [17]. These biases can be reduced by the following four methods: (1) exclusion of deaths or episodes occurring within the first 7 days of life; (2) exclusion of infants and young children from non-singleton and/or premature births and those with low birth weight, congenital abnormalities, and any other serious illnesses unrelated to the outcome of interest; (3) identification of breastfeeding exposure immediately *prior* to the onset of illness or mortality as opposed to that concurrent with outcome; (4) assessment of whether weaning was a direct consequence of illness or poor growth and exclusion of such infants or young children if their inclusion significantly changes the effect measure [18]. Under our scoring system, we assigned a study 0.5-1 point for failure to incorporate each of these four methods, such that reverse causality was considered not likely, likely, and highly likely for studies with zero, 0.5-2 and 2.5-4 points, respectively. The studies and the data extracted from each as well as details on scoring studies for reverse causality are available in additional file 1.

## Results

**Figure 1:**
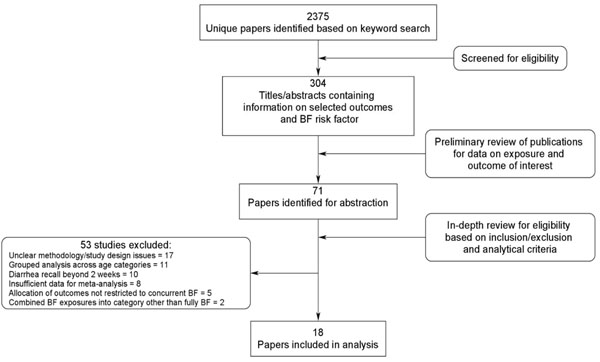
Synthesis of study identification in review process of the effects of suboptimal breastfeeding exposure on diarrhea incidence, prevalence, mortality, hospitalizations, and all-cause mortality

The systematic literature review yielded 2375 unique publications, 71 of which contained data on suboptimal breastfeeding as a risk factor for the identified outcomes of interest (Figure 1). A total of 18 studies met all inclusion, exclusion, and analytical criteria and were included in the analysis [15,19-35]. Of these, 11 were prospective cohort, 4 were cross-sectional observational, and 3 were case-control studies. The majority were conducted in Latin America (n=7) but also took place in Africa (n=4), South Asia (n=5), the Middle East (n=2) and the Western Pacific (n=2) regions, with one study reporting three different study locations. The numbers of studies included in each meta-analysis are listed in Tables 2, 3, 4.

**Table 2:** The effect of suboptimal breastfeeding on selected outcomes during infancy

		0-5 months*			0-11 months*	
**Outcome**	**Reference Category**	**Predominant**	**Partial**	**Not**	**Partial**	**Not**

Diarrhea Incidence	Exclusive	1.26 (0.81-1.95)[22]	1.68 (1.03-2.76)[22,23,28]	2.65 (1.72-4.07)[22,23,28]		
	Predominant		1.77 (0.82-3.83)[22,26,27]	2.08 (1.58-2.72)[22,27]		
	Partial			1.71 (1.38-2.11)[22,23,27]		

						

Diarrhea Prevalence	Exclusive	2.15 (1.81-2.55)[22,30,32,34]	4.62 (2.37-9.00)[22,30,32]	4.90 (2.93-8.21)[22,32,34]		
	Predominant		1.46 (0.95-2.26)[22,27,30]	2.40 (1.31-4.43)[22,27,34]		
	Partial			2.05 (1.46-2.88)[22,27]		
	Any					1.21 (0.95-1.53)[34]

						

Diarrhea Mortality	Exclusive	2.28 (0.85-6.13)[19,20]	4.62 (1.81-11.76)[19,20]	10.52 (2.79-39.6)[19,20]		
	Predominant		2.41 (1.21-4.83)[20]	7.88 (2.64-23.46)[20]	4.19 (2.24-7.84)[25,33]	11.73 (4.71-29.21)[25,33]
	Partial			3.26 (1.15-9.25)[20]		1.69 (1.11-2.58)[25]

						

All-Cause Mortality	Exclusive	1.48 (1.14-1.92)[19,20,24]	2.84 (1.63-4.97)[19,20,24]	14.40 (6.13-33.86)[19,20]		
	Predominant		1.69 (1.10-2.61)[20]	8.08 (4.45-14.69)[20]		
	Partial			4.77 (2.65-8.61)[20]		

						

Diarrhea Hospitalization	Exclusive	2.28 (0.08-6.55)[20]	4.43 (1.75-13.84)[20]	19.48 (6.04-62.87)[20]		
	Predominant		3.16 (1.42-7.05)[20,29]	16.41 (4.59-58.69)[20,29]		
	Partial			3.95 (1.91-8.19)[20]		

*Effect reported as RR (95% CI)^[Ref]^

**Table 3:** The effect of not breastfeeding on selected outcomes in children 6-23 months of age

	6-11 months*	6-23 months*	12-23 months*
**Outcome**			

Diarrhea Incidence	1.32 (1.06-1.63)[22,27]	-	-

			

Diarrhea Prevalence	2.63 (1.04-6.65)[22,27,31]	2.07 (1.49-2.88)[21,22,27,31]	1.39 (1.07-1.80)[21,31]

			

Diarrhea Mortality	1.47 (0.67-3.25)[19,35]	2.18 (1.14-4.16)[15,19,35]	2.57 (1.10-6.01)[15,35]

			

All-Cause Mortality	5.66 (1.86-17.20)[19]	3.69 (1.49-9.17)[19,21]	2.23 (0.65-7.59)[21]

			

Diarrhea Hospitalization	6.05 (2.44-14.97)[29]	-	-

*Effect reported as RR (95% CI)^[Ref]^; Any breastfeeding is reference category

**Table 4:** The effect of suboptimal breastfeeding on selected outcomes in neonates

				
**Outcome**	**Reference Category**	**Predominant**	**Partial**	**Not**

Diarrhea Incidence	Exclusive	-		
	Predominant		1.67 (0.50-5.52)[27]	0.69 (0.09-5.49)[27]
	Partial			0.41 (0.05-3.68)[27]

				

Diarrhea Prevalence	Exclusive			
	Predominant		4.44 (2.42-8.16)[27]	1.83 (0.73-4.60[27]
	Partial			0.41 (0.17-1.00)[27]

				

Diarrhea Mortality	Exclusive			
	Predominant		1.40 (0.13-15.42)[19]	
	Partial			

				

All-Cause Mortality	Exclusive	1.41 (1.00-1.99)[19,24]	2.96 (0.75-11.69)[19,24]	1.75 (0.30-10.26)[19]
	Predominant		1.33 (0.61-2.91)[19]	1.94 (0.59-6.43)[19]
	Partial			1.46 (0.40-5.29)[19]

*Effect reported as RR (95% CI)^[Ref]^

### Diarrhea incidence

**Figure 2:**
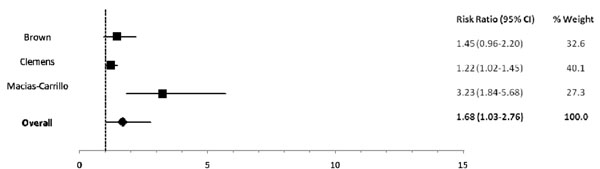
Forest plot for the effect of partial breastfeeding as compared to exclusive breastfeeding on diarrhea incidence among infants 0-5 months of age

**Figure 3:**
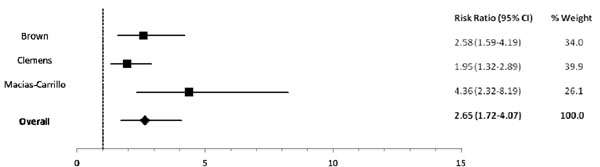
Forest plot for the effect of not breastfeeding as compared to exclusive breastfeeding on diarrhea incidence among infants 0-5 months of age

**Figure 4:**
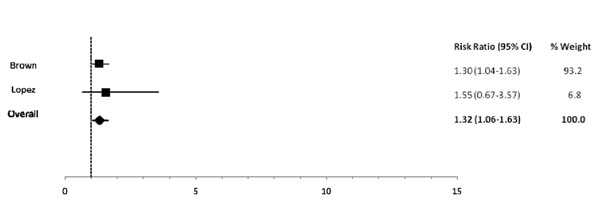
Forest plot for the effect of not breastfeeding as compared to any breastfeeding on diarrhea incidence among infants 6-11 months of age

Among infants 0-5 mos of age (Table 2), predominant (RR: 1.26), partial (RR: 1.68) and not breastfeeding (RR: 2.65) resulted in an excess risk of incident diarrhea in comparison to exclusive breastfeeding (Figures 2, 3). Similarly, the estimated relative risk of incident diarrhea was elevated when comparing not breastfed (RR: 1.32) to breastfed infants 6-11 mos of age (Table 3; Figure 4). No studies reported diarrhea incidence comparing exclusive breastfeeding to suboptimal feeding among neonates.

### Diarrhea prevalence

In comparison to exclusively breastfed infants 0-5 mos of age, the estimated relative risk of prevalent diarrhea was statistically significantly elevated in predominantly (RR: 2.15), partially (RR: 4.62), and not (RR: 4.90) breastfed infants (Table 2). Among infants and young children 6-23 mos of age (Table 3), not breastfeeding (RR: 2.07) resulted in an excess risk of prevalent diarrhea as compared to breastfeeding. There were no studies comparing diarrhea prevalence among exclusively and suboptimally breastfed neonates (Table 4).

### Diarrhea mortality

**Figure 5:**
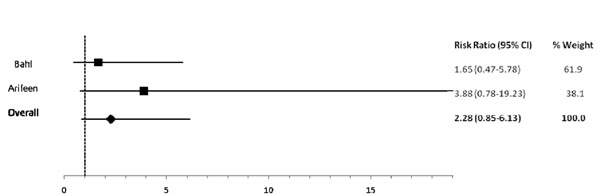
Forest plot for the effect of predominant breastfeeding as compared to exclusive breastfeeding on diarrhea mortality among infants 0-5 months of age

**Figure 6:**
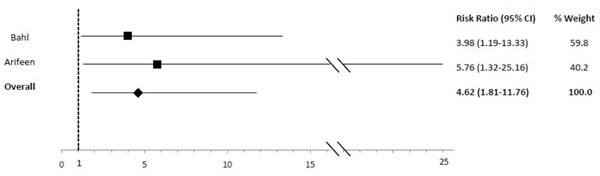
Forest plot for the effect of partial breastfeeding as compared to exclusive breastfeeding on diarrhea mortality among infants 0-5 months of age

**Figure 7:**
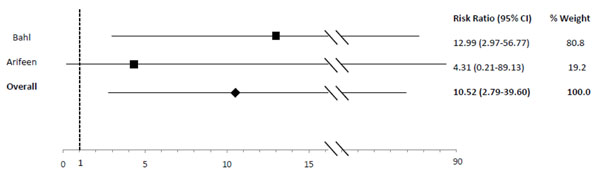
Forest plot for the effect of not breastfeeding as compared to exclusive breastfeeding on diarrhea mortality among infants 0-5 months of age

**Figure 8:**
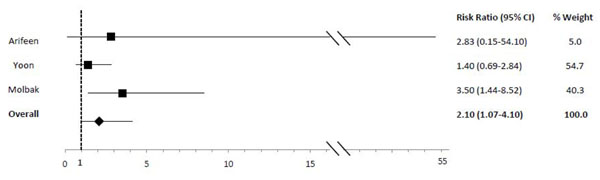
Forest plot for the effect of not breastfeeding as compared to any breastfeeding on diarrhea mortality among infants 6-23 months of age

In comparison to exclusive breastfeeding, predominant (RR: 2.28), partial (RR: 4.62) and not (RR: 10.52) breastfeeding led to an elevated risk of diarrhea mortality among infants 0-5 mos of age (Table 2; Figures 5, 6, 7). Among infants 0-11 mos of age (Table 2), the estimated risk of diarrhea mortality was higher in partially (RR: 4.19) and not (RR: 11.73) breastfed infants as compared to those predominantly breastfed. For infants and young children 6-23 mos of age (Table 3), not breastfeeding (RR: 2.18) resulted in an excess risk of diarrhea mortality as compared to breastfeeding (Figure 8). There were no studies comparing the outcome of diarrhea mortality in exclusively versus suboptimally breastfed neonates (Table 4).

### All-cause mortality

As compared to exclusively breastfed infants 0-5 mos of age (Table 2), the estimated relative risk of all-cause mortality was statistically significantly elevated among those predominantly (RR: 1.48), partially (RR: 2.84) and not (RR: 14.40) breastfed. The estimated relative risk of all-cause mortality was higher when comparing not breastfed (RR: 3.69) to breastfed infants and young children 6-23 mos of age (Table 3). Among neonates, predominant (RR: 1.41), partial (RR: 2.96), and no (RR: 1.75) breastfeeding resulted in elevated risk of mortality as compared to exclusive breastfeeding (Table 4).

### Diarrhea hospitalizations

The estimated relative risk of hospitalization for diarrhea illness was elevated among predominantly (RR: 2.28), partially (RR: 4.43) and not (RR: 19.48) breastfed infants 0-5 mos of age as compared to those exclusively breastfed (Table 2). Among infants 6-11 mos of age (Table 3), not breastfeeding continued to result in a higher risk of hospitalization for diarrhea when compared to any breastfeeding (RR: 6.05). There were no studies reporting diarrhea hospitalizations as an outcome for neonates (Table 4).

### Quality assessment and effect size estimates for LiST

In table 5, we report the quality assessment of studies by outcome. Using the CHERG grading system for study design and study quality [11], outcome-specific quality was moderate for all outcomes of interest. Although reverse causation bias was likely or highly likely in the majority of studies, outcome-specific findings were largely consistent with all but two studies confirming the highly protective effect of exclusive breastfeeding and any breastfeeding among infants 0-5 mos of age and young children 6-23 mos of age, respectively.

**Table 5:** Quality assessment of studies measuring the association between suboptimal breastfeeding and selected outcomes

				Directness	
				
No of studies ^(ref)^	Design	Limitations	Consistency	Generalizability to population of interest	Generalizability to intervention of interest
***Diarrhea Incidence: moderate outcome-specific quality***					

5 [22,23,26-28]	Cohort/Cross-sectional	Reverse causality highly likely or likely for all 5 studies (-0.5)	Consistent and all studies showing benefit of EBF among infants 0-5 mos of age and benefit of any BF among children 6-23 mos of age (+1)	Mostly Latin America (-0.5)	EBF not reported for neonates alone

***Diarrhea Prevalence (1-2 week): moderate outcome-specific quality***					

7 [21,22,27,30-32,34]	Cohort/Cross-sectional	Reverse causality highly likely or likely for all 7 studies (-0.5)	All but one study showing benefit of EBF among infants 0-5 mos of age; all studies showing benefit of any BF among children 6-23 mos of age (+1)	Mostly Asia (-0.5)	EBF not reported for neonates alone

***Diarrhea Mortality: moderate outcome-specific quality***					

6 [15,19,20,25,33,35]	Cohort/Case-control	Reverse causality highly likely or likely for 5 of 6 studies (-0.5)	Consistent and all studies showing benefit of EBF among infants 0-5 mos of age and benefit of any BF among children 6-23 mos of age (+1)	Mostly Asia & Latin America (-0.5)	EBF not reported for neonates alone

***All-Cause Mortality: moderate outcome-specific quality***					

4 [19-21,24]	Cohort	Reverse causality highly likely or likely for all 4 studies (-0.5)	All but one study showing benefit of EBF among infants 0-5 mos of age; all studies showing benefit of any BF among children 6-23 mos of age (+1)	Mostly Asia (-0.5)	

***Diarrhea Hospitalizations: moderate outcome-specific quality***					

2 [20,29]	Cohort/Case-control	Reverse causality highly likely or likely for both studies (-0.5)	Consistent and all studies showing benefit of EBF among infants 0-5 mos of age and benefit of any BF among children 6-23 mos of age (+1)	Equal amount of data from Asia, Latin America, Africa & Eastern Mediterranean	EBF not reported for neonates alone

Applying the CHERG standard rules, strong evidence exists for the reduction of diarrhea incidence and diarrhea mortality by exclusive breastfeeding among infants 0-5 mos of age and by any breastfeeding among children 6-23 mos of age. In table 6, we present the final effect size estimates to be entered into LiST.

**Table 6:** Application of standardized rules for choice of final outcome to estimate effect of breastfeeding on the reduction of diarrhea mortality

Outcome Measures		Application of Standard Rules
0-5 months*		
**Diarrhea Incidence**	**n=3; 1594 events** The risk of incident diarrhea is 1.26 (0.81-1.95) for predominant BF; 1.68 (1.03-2.76) for partial BF; 2.65 (1.72-4.07) for not BF as compared to EBF	**Rule 2: APPLY**
**Diarrhea Mortality**	**n=2; 80 events** The risk of diarrhea mortality is 2.28 (0.85-6.13) for predominant BF; 4.62 (1.81-11.76) for partial BF; 10.52 (2.79-39.6) for not BF as compared to EBF	

**6-11 months**		
**Diarrhea Incidence**	**n=2; 646 events** The risk of incident diarrhea is 1.32 (1.06-1.63) for not BF as compared to any BF	**Rule 2: APPLY**
**Diarrhea Mortality**	**n=2; 84 events** The risk of diarrhea mortality is 1.47 (0.67-3.25) for not BF as compared to any BF	

**12-23 months**		
**Diarrhea Incidence**	**n=0; use estimate for 6-11 mos: n=2; 646 events** The risk of incident diarrhea is 1.32 (1.06-1.63) for not BF as compared to any BF	**Rule 2: APPLY**
**Diarrhea Mortality**	**n=2; 84 events** The risk of diarrhea mortality is 2.57 (1.10-6.01) for not BF as compared to any BF	

*Evaluating events for studies where reference category is EBF

## Discussion

We found a sizable body of evidence for the protective effects of breastfeeding against diarrhea incidence, prevalence, hospitalizations, diarrhea mortality, and all-cause mortality. The results of random effects meta-analyses of eighteen included studies indicated varying degrees of protection across levels of breastfeeding exposure [15,19-35] .

For all outcomes among infants 0-5 mos of age, the protection conferred by exclusive breastfeeding was incrementally greater than that granted by predominant and partial breastfeeding (Table 2). Our results also confirmed a protective effect of any breastfeeding against all outcomes among infants 6-23 mos of age. The data for neonates alone are limited in that comparisons to exclusive breastfeeding, the WHO recommendation for this age group, were not reported for four out of the five identified outcomes of interest. Overall, our estimated effect sizes were large, thus suggesting a protective effect of breastfeeding among neonates.

The protection conferred by breastfeeding appears to operate via two pathways, decreasing diarrhea incidence as well as duration. The effect sizes appear to be larger for the reduction of diarrhea prevalence as compared to incidence suggesting that the predominate mechanism by which breastfeeding reduces diarrhea mortality is through the reduction of prolonged episodes.

In comparison to the Lancet nutrition series [7], we report effect estimates for two additional outcomes—diarrhea prevalence and diarrhea hospitalizations, as well as additional estimates for neonates separate from the 0-5 months age category. We also conducted meta-analyses comparing reference groups other than exclusive breastfeeding for infants 0-5 months of age. The results of our systematic review closely mirrored the final data set included in the Lancet nutrition series and thus report nearly identical effect estimates for the meta-analyses of all-cause mortality for 0-5 mos and 6-23 mos; diarrhea mortality for 0-5 mos; and diarrhea incidence for predominantly compared to exclusively breastfed infants 0-5 mos of age. We excluded two previously included studies on the basis of diarrhea recall beyond two weeks [36,37], and we included four additional studies not cited by the Lancet nutrition series [15,23,28,35]. This resulted in lower effect estimates than those previously reported for the risk of diarrhea mortality and incidence in not breastfed children 6-23 mos of age and for the risk of incident diarrhea in partially and not breastfed infants 0-5 mos of age. Although we included one of the three studies included by the Lancet nutrition series in the estimation of the risk of diarrhea incidence among children 6-23 mos [22], we further stratified our results in this age category and thus report this RR under 6-11 rather than 6-23 mos. Overall, our results confirm and expand upon the protective effects of breastfeeding as previously reported by the Lancet nutrition series.

Although the majority of studies included in this review did not methodologically account for the possibility of reverse causation, it is highly unlikely that this potential bias was responsible for the large effect sizes and consistent findings observed across all age categories and outcomes. This assertion is evidenced by the comparability of findings before and after adjusting for reverse causality within included studies [4,18,20,24]. Repeat analyses excluding all deaths occurring within 7 days of a feeding assessment did not statistically significantly alter the effect measures observed by Bahl et al [20]. Similarly, the adjusted odds ratio (2.40; 95% CI: 1.69-3.40) reported by Edmond et al. was very similar to the ORs observed after excluding infants dying within the first week of life (OR: 2.36; 95% CI: 1.44-3.87) or those at high risk of death due to premature birth, congenital anomaly, or ill health at the time of interview (OR: 2.44; 95% CI: 1.60-3.74) [24]. Despite observing substantially higher relative risks before methodologically accounting for reverse causality, the strong protective effect of breastfeeding noted by Victora et al. persisted following this adjustment [4,18].

While the current analysis was limited by a lack of geographic variety by outcome, the geographic diversity of the overall analysis was actually quite wide with studies taking place in eleven unique countries and in all WHO regions except Europe.

Additionally, the current analysis was limited in that effect measures from studies publishing raw data or estimates in a form insufficient for meta-analysis were computed without correcting for potential confounders to breastfeeding exposure, such as socioeconomic status. Still, we do not expect this to constitute a major limitation since similar methodology has been used in previous studies and since the direction and magnitude of effect sizes were consistent when comparing studies with and without controls for confounding. Furthermore, lack of adjustment for confounding may have actually led to an underestimation of the protective effect of breastfeeding, since poverty is associated with longer breastfeeding duration in many of the developing country populations included in this analysis [38].

The quality assessment resulted in a score of moderate outcome-specific quality (Table 5). According to CHERG standards, the overall score of moderate quality across all outcomes of this analysis indicates that these data represent the best available estimate of the protective effect of breastfeeding against diarrhea-specific morbidity and mortality and can therefore be included in the LiST model with confidence [11].

WHO and UNICEF currently recommend exclusive breastfeeding for the first 6 mos of life with continued feeding through the first year among HIV positive mothers, provided that they or their infants receive ARV drugs during the breastfeeding period.[39] In this review we did not attempt to quantify the relative risks of alternative infant feeding practices in HIV positive populations. Though there are numerous studies suggesting that exclusive breastfeeding during the first 6 mos and continued breastfeeding for the second 6 mos decrease mortality among infants born to HIV positive mothers [40,41], further research is warranted as to whether the effect sizes reported here are relevant among HIV positive mothers and infants.

## Conclusions

In conclusion, our data confirm and highlight the importance of breastfeeding for the prevention of diarrhea morbidity and mortality. This review also provides updated risk estimates across age categories. Among infants 0-5 mos of age, these findings support the recommendation for exclusive breastfeeding during the first 6 months of life as a key child survival intervention. Furthermore, results among infants and children beyond the first 6 mos of age reveal the importance of continued breastfeeding as a critical intervention to protect against diarrhea-specific morbidity and mortality throughout the first two years of life. Though we have confidence in the strength of the evidence presented here, continued research will be needed to update the effect size estimates as diarrhea and all cause mortality rates continue to decline in many developing countries.

This review does not evaluate the effect of breastfeeding promotion strategies or the operational challenges of inspiring mothers to commit to exclusive breastfeeding for the first 6 months and to continued breastfeeding for the following 18 months. Operations research is needed to identify methods for maximizing the effectiveness of breastfeeding promotion programs and policies on behaviour change among mothers.

## Competing interests

The authors have no competing interests.

## Authors' contributions

LML conducted the systematic review, analysis and led the initial manuscript preparation. CLFW assisted with the analysis and the manuscript preparation. AN contributed to the systematic review, data abstraction, and manuscript preparation. CV and REB provided technical leadership and assisted with the interpretation of the analysis and the final manuscript preparation.

## Supplementary Material

Additional file 1 **Breastfeeding and Diarrhea** The additional file 1 is an excel spreadsheet named Final_Web_Appendix. This file contains two sheets. The ‘Data Abstraction’ sheet includes all data abstracted from studies, as well as notes on methodology and limitations. The ‘Reverse Causality’ sheet includes the assessment sheet used to systematically score studies on reverse causation bias. Click here for file
